# Dihydromyricetin attenuates palmitic acid-induced oxidative stress by promoting autophagy via SIRT3-ATG4B signaling in hepatocytes

**DOI:** 10.1186/s12986-021-00612-w

**Published:** 2021-09-09

**Authors:** Li Huang, Xianglong Zeng, Bo Li, Cong Wang, Min Zhou, Hedong Lang, Long Yi, Mantian Mi

**Affiliations:** 1 Research Center for Nutrition and Food Safety, Chongqing Key Laboratory of Nutrition and Food Safety, Chongqing Medical Nutrition Research Center, Institute of Military Preventive Medicine, Third Military Medical University, 30th Gaotanyan Main Street, Shapingba District, 400038 Chongqing, People’s Republic of China; 2 General Hospital of Tibet Military Command Area, 850000 Lhasa, Tibet People’s Republic of China; 3 Department of Blood Transfusion, 925 Hospital, Joint Logistics Support Force, PLA, 550009 Guiyang, People’s Republic of China

**Keywords:** Dihydromyricetin, NASH, Oxidative stress, Autophagy, ATG4B, Mitochondria, SIRT3

## Abstract

**Background:**

Oxidative stress in hepatocytes was important pathogenesis of nonalcoholic steatohepatitis (NASH). Autophagy was a cellular process that can remove damaged organelles under oxidative stress, and thus presented a potential therapeutic target against NASH. This work aimed to investigate whether autophagy was participated in the protective effects of dihydromyricetin (DHM) on palmitic acid (PA)-induced oxidative stress in hepatocytes and the underlying mechanism.

**Methods:**

HepG2 and HHL-5 cell lines were pretreated with DHM (20 μM) for 2 h, followed by PA (0.2 mM) treatment for 16 h. The oxidative stress was assessed by the quantification of intracellular reactive oxygen species (ROS), mitochondrial ROS (mtROS), mitochondrial membrane potential (MMP) and mitochondrial ultrastructural analyses. The protein expressions of SIRT3, LC3I/II, P62 and ATG4B, as well as the acetylation of AGT4B were determined by western blotting using HepG2 and HepG2/ATG4B^±^ cells with heterozygous knockout of *ATG4B*.

**Results:**

Exposure to PA resulted in increased intracellular ROS and mtROS, decreased MMP and aggravated mitochondrial injury in HepG2 cells, which were notably attenuated by DHM treatment. DHM-induced inhibition of oxidative stress was associated with the induction of autophagy, characterized by upregulated ATG4B and LC3 II as well as downregulated P62 levels. Furthermore, the inhibitory effects of DHM on PA-induced autophagy arrest and oxidative stress were eliminated when pretreated with a SIRT3 inhibitor 3-TYP or conducted in HepG2/ATG4B^±^ cells, suggesting that SIRT3 and ATG4B were involved in DHM-induced benefits. Moreover, DHM treatment increased the protein expression of SIRT3 and SIRT3-dependent deacetylation of ATG4B in HepG2 cells.

**Conclusion:**

Our results demonstrated that DHM attenuated PA-induced oxidative stress in hepatocytes through induction of autophagy, which was mediated through the increased expression of SIRT3 and SIRT3-mediated ATG4B deacetylation following DHM treatment.

**Supplementary Information:**

The online version contains supplementary material available at 10.1186/s12986-021-00612-w.

## Introduction

Nonalcoholic fatty liver disease (NAFLD) is a spectrum of liver disease which is becoming the main cause of chronic liver disease. The global prevalence of NAFLD is approximately 25%, ranging from 13% in Africa1 to 42% in southeast Asia [1]. Nonalcoholic steatohepatitis (NASH) is believed to be a more advanced liver pathology, which could progress to hepatic fibrosis, cirrhosis and even hepatocellular carcinoma (HCC). NASH prevalence is projected to increase by up to 56% between 2016 and 2030 in China, the USA, Western Europe and Japan [2]. According to the “two-hit theory”, liver steatosis (the “first hit”) induces fat accumulation, followed by oxidative stress (the “second hit”) which leads to proinflammatory molecules release, mitochondrial damage and lipid peroxidation [3]. The consequential surplus of lipids in hepatocytes leads to lipotoxicity and oxidative stress, which promotes mitochondrial dysfunction through multiple mechanisms including augmented reactive oxygen species (ROS) production and decreased mitochondrial membrane potential (MMP) [4]. Much evidence suggests that oxidative stress, which promotes mitochondrial dysfunction especially in hepatocytes, represents a key element in driving hepatic inflammation in NASH, leading to hepatocyte dysfunction or death [5]. Palmitic Acid (PA), which was commonly employed for NAFLD induces oxidative stress by endoplasmic reticulum (ER) stress, ER calcium depletion, intracellular calcium dyshomeostasis and mitochondrial dysfunction, respectively [6–8]. Thus, protecting hepatocytes against oxidative stress and maintaining redox homeostasis is important to reverse or retard the progression of NASH.

Autophagy, a conserved cellular process, can reduce oxidative stress by degrading cytoplasm and damaged organelles, such as mitochondria, through lysosomes or vacuoles to maintain cellular environment homeostasis [9]. Much evidence indicates that NASH is accompanied by decreased autophagy and increased oxidative stress [10–12]. Autophagy-related 4B cysteine peptidase (ATG4B) is the most active protease among the four mammalian paralogs of ATG4, which acts an important role in the regulation of autophagy [13, 14]. Evidence indicates that ATG4B can be acetylated at the site of N-terminus, which is one of the major protein modifications in eukaryotes [15]. Up to date, the underlying mechanisms of ATG4B and its acetylation in the development and progression of NASH remains unknown.

SIRT3, a member of the mammalian sirtuin family protein that is localized to mitochondria, has robust deacetylase activity and plays an essential role in the regulation of metabolic homeostasis and energy metabolism [16]. With dysregulated protein acetylation in the mitochondria, *SIRT3* knockout mice fed with high-fat diet exhibited aggravated metabolic syndrome [17]. And overexpression of *SIRT3* preserved electron transport chain, enhanced adenosine triphosphate generation and strengthened autophagy [16, 18–20]. However, a recent study demonstrated that overexpression of SIRT3 inhibited adenosine monophosphate-activated protein kinase and rapamycin C1 activation, leading to autophagy suppression [21]. These differing results raise the question that whether SIRT3 could be a critical regulator of autophagy in NASH.

Dihydromyricetin (DHM) is a natural flavonoid compound that mainly exists in *Ampelopsis grossedentata* [22]. In our previous study, DHM supplementation promoted lipid metabolism in NAFLD patients [23], attenuated hepatic steatosis and maintained redox homeostasis through increased SIRT3 expression [24]. Previous researches have shown that DHM could induce autophagy in HepG2 cells [25–27]. Thus, we hypothesized that DHM might attenuate oxidative stress by regulating autophagy in hepatocytes. As expected, these data provided new evidence that DHM significantly inhibited oxidative stress in hepatocytes through induction of autophagy via SIRT3-ATG4B signaling pathway.

## Materials and methods

### Reagents

DHM (CAS No. 27200-12-0, HPLC ≥ 98%) was obtained from Chengdu Mansite Bio-Technology (Chengdu, China). Fetal bovine serum (FBS) and Dulbecco’s modified Eagle’s medium (DMEM) were purchased from Gibco (Carlsbad, CA, USA). Dimethyl sulfoxide (DMSO) and palmitic acid (PA) were obtained from Sigma-Aldrich (St. Louis, MO, USA). Penicillin/streptomycin and bovine serum albumin (BSA) were obtained from Beyotime (Shanghai, China). Antibodies against acetyl-lysine (AcK), voltage-dependent anion channel (VDAC), P62 and LC3 were purchased from Cell Signaling Technology (Beverly, MA, USA). Antibodies against beta-actin (ACTB), ATG4B, and SIRT3 were obtained from Abcam (Cambridge, MA, USA). Antibody against optic atrophy 1 protein (OPA1) was purchased from BD Biosciences (San Jose, CA, USA). Chloroquine (CQ), 3-methyladenine (3-MA) and 3-(1H-1, 2, 3-triazol-4-yl) pyridine (3-TYP, CAS No. 120241–79-4) were purchased from MedChemExpress (Monmouth Junction, NJ, USA). Other reagents were purchased as indicated in the corresponding methods.

### Cell culture and treatments

HepG2 cell line was provided from the American Type Culture Collection (Manassas, VA, USA). HepG2/ATG4B^±^ cell line, in which *ATG4B* was heterozygous knockout with CRISPR-Cas9 system, was a gift from Nan Zhang (Department of Biochemistry and Molecular Biology, College of Basic Medical Sciences, Third Military Medical University, Chongqing 400038, China) [28]. HHL-5 cell line gifted by Professor Arvind H Patel from the Virus Research Center of the University of Glasgow. All cell lines were cultured in DMEM medium with 10% FBS, 1% penicillin/streptomycin at 37 °C and 5% CO_2_ incubator, and cells from the 3rd to 6th passages were used for the experiments. To detect the effect of DHM on PA-induced oxidative stress in hepatocytes, both cell lines were pretreated with DHM (20 μM) or the vehicle (0.5% DMSO) for 2 h before the treatment of PA (0.2 mM) for an additional 16 h as described previously [24]. To determine the involvement of autophagy and SIRT3 in DHM-induced inhibition of oxidative stress in hepatocytes, the autophagy inhibitor 3-MA (0.5 mM) and CQ (20 μM), as well as a SIRT3 inhibitor 3-TYP (50 μM) were used 1 h prior to DHM treatment, respectively.

### Green fluorescent protein (GFP)-LC3 transfection

After seeded on cell culture dish (NEST, China) for 8 h, HepG2 cells were transfected with GFP-LC3 expression vector (kindly provided by MengYu Liu, Department of Occupational Health, Third Military Medical University, Chongqing 400,038, China) at the final concentration of 1 μg/μL for 4 h using Lipofectamine 2000 (Invitrogen, Carlsbad, CA) according to the manufacturer’s instructions. Samples were then analyzed using laser confocal scanning microscopy (Zeiss, Germany).

### Immunofluorescence microscopy analysis

HepG2 cells were loaded with MitoTracker Red (500 nM, Cell Signaling Technology, USA) to label the mitochondria as described [29]. Briefly, after treated as indicated, the HepG2 cells were loaded with MitoTracker Red and imaged by laser confocal scanning microscopy (Zeiss, Germany) under the excitation (Ex) and emission (Em) wavelength of 644 nm and 665 nm, respectively. To observe the mitochondrial fusion, HepG2 cells were labeled with MitoTracker Red and then fixed with 4% paraformaldehyde (Beyotime, China), permeabilized with 0.25% Triton X-100 (Beyotime, China) and blocked with 5% goat serum (Beyotime, China). Then, cells were incubated with primary antibody against OPA1 at 4 °C overnight and incubated with Alexa Fluor® 488 goat anti-rabbit IgG secondary antibody (1:200, Beyotime, China). Finally, nuclei were counterstained with DAPI. Samples were analyzed using laser confocal scanning microscopy (Zeiss, Germany).

### Intracellular and mitochondrial ROS assessment

The intracellular and mitochondrial ROS (mtROS) levels were determined using DCFH-DA (Beyotime, China) and MitoSOX™ Red (Invitrogen, Carlsbad, CA) according to the manufacturer’s instructions, respectively. After treated as indicated, cells were loaded with DCFH‐DA (10 μM) in FBS-free DMEM at 37 °C for 30 min, then washed twice with FBS-free DMEM to detect the intracellular ROS level. Meanwhile, cells were loaded with MitoSOX™ Red (5 μM) at 37 °C for 30 min, then washed gently three times with warm PBS to detect the mtROS level. The mean fluorescence intensity of intracellular ROS (Ex/Em: 488/525 nm) and mtROS (Ex/Em: 510/580 nm) were determined using flow cytometry (FCM; Accuri C6, BD Biosciences, San Jose, CA), respectively.

### MMP measurement

The MMP was measured using JC-1 probe (Beyotime, China, CAS No. 47729-63-5) according to the manufacturer’s instruction. Briefly, after treated as indicated, cells were loaded with JC-1, followed by washing twice in PBS before being subjected to FCM for green fluorescence (Ex/Em: 488/525 nm) and red fluorescence (Ex/Em: 525/590 nm) (Accuri C6, BD Biosciences, San Jose, CA). Fluorescence intensity ratio was calculated to reflect the MMP.

### Transmission electron microscopy (TEM)

The mitochondrial morphology was observed by TEM. Briefly, HepG2 cells were treated as described previously [29] and visualized on TEM (EM420, Amsterdam, The Netherlands). Three samples per group and ten images of each sample were selected for quantitative analysis. Any types of ultrastructural changes including broken inner and outer membranes, intramitochondrial edema, disrupted cristae, hypertrophic giant mitochondria as well as intramitochondrial edema were considered damaged and dysfunctional mitochondria. The ratio of the number of normal mitochondria to the total was calculated to evaluate the percentage of normal mitochondria.

### Western blotting and co-immunoprecipitation (Co-IP)

Cells were lysed in cell lysis buffer (Sigma-Aldrich, St. Louis, MO, USA) containing 1% protease inhibitor cocktail and phosphatase inhibitor cocktail (Roche, Basel, Switzerland). The mitochondrial proteins were extracted using a Cell Mitochondria Isolation Kit (Beyotime, China) with protein concentrations determined using a BCA kit (Beyotime, China), and samples mixed with loading buffer (Beyotime, China) were heated at 100 °C for 10 min. For Western blotting, equal amounts of proteins were resolved via SDS-PAGE (10–15%), followed by transfer and fixation on polyvinylidene difluoride membranes (0.22 μm; Millipore, Billerica, MA, USA). The membranes were blocked with 5% skimmed milk for 2 h and then immunoblotted with antibodies (1:1000) overnight at 4 °C. Then, the membranes were washed with tris-buffered saline with Tween (TBST) buffer three times, followed by the incubation with the appropriate secondary antibody (Sigma-Aldrich, St. Louis, MO, USA) (1:5000) for 1 h and then washing with TBST buffer three times. The immunostained bands were visualized and determined using Fusion FX (Vilber Lourmat, France) and Immobilon Western Chemiluminescent HRP Substrate (Millipore, USA). ACTB and VDAC were used as loading controls for whole cell lysates and mitochondrial lysates, respectively. Densitometry analysis was carried out using ImageJ software. For Co-IP analysis, the proteins were diluted in lysis buffer and then mixed with the corresponding antibodies as well as protein A/G-agarose bead slurry (Cell Signaling Technology, Beverly, MA, USA) overnight at 4 °C with gentle rotation. Then, the mixtures were centrifuged at 1100×*g* for 3 min at 4 °C and washed with lysis buffer five times followed by Western blotting for immunoblotting. The specificity of antibodies used for immunoprecipitation was routinely validated by running negative controls with non-immune IgG under the same conditions.

### Statistical analysis

Data analysis was performed with SPSS 19.0 software (Chicago, IL, USA). All experimental data were expressed as mean ± SD of at least 3 experiments. Statistical differences among groups were determined with either Student’s t-test (for two groups) or one-way analysis of variance (ANOVA) followed by LSD post hoc tests (for multiple group comparisons). A p value less than 0.05 was considered to indicate a statistically significant difference; ns means not significant.

## Results

### DHM inhibits PA-induced oxidative stress in hepatocytes

**Fig. 1:**
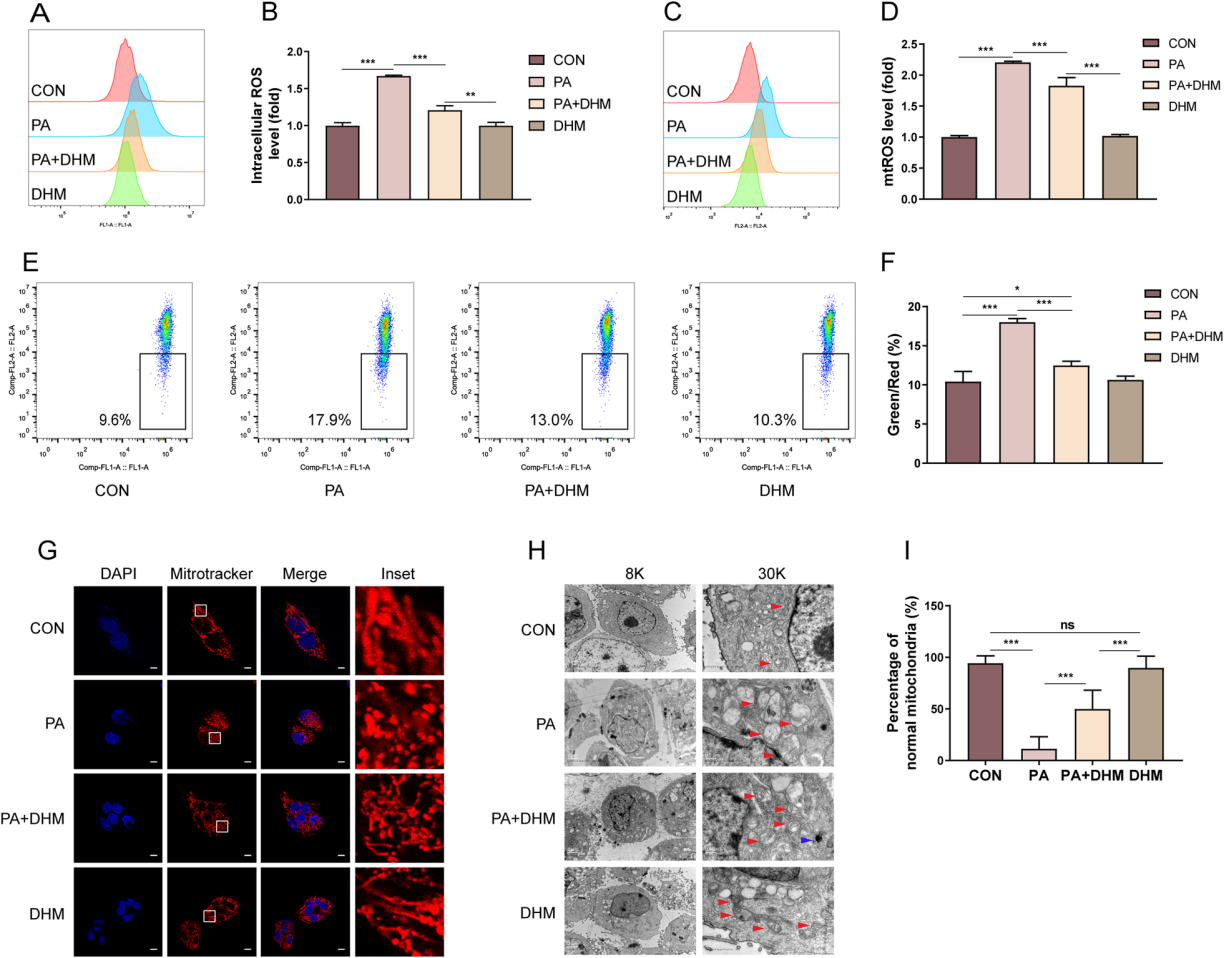
DHM inhibits PA-induced oxidative stress in hepatocytes. HepG2 cells were pretreated with 20 μM of DHM or the vehicle (0.5% DMSO) for 2 h, followed by the treatment of 0.2 mM of PA for an additional 16 h. **A**, **B** HepG2 cells were labeled with DCFH-DA probe and the intracellular ROS levels were measured by FCM assay. **C**, **D** HepG2 cells were labeled with MitoSOX™ Red probe and the mitochondria ROS (mtROS) levels were quantified by FCM assay. **E**, **F** HepG2 cells were labeled with JC-1 probe and the MMP level was quantified by FCM assay. **G** HepG2 cells were stained with MitoTracker Red and counterstained with DAPI. Representative images were acquired by a laser scanning confocal microscope, scale bars: 10 μm. **H** Representative TEM images of HepG2 cells at a magnification of ×8000 (left) and ×30,000 (right). The red arrows indicate mitochondria, and the blue arrow indicates an autophagosome. **I** The percentage of normal mitochondria in TEM images. Graphs showed mean ± SEM; n = 3; data were from one experiment out of three. *p < 0.05, **p < 0.01, ***p < 0.001, compared between the marked groups; *ns* no significant difference

To clarify the effects of DHM on PA-induced oxidative stress in hepatocytes, HepG2 cells were labeled with DCFH-DA probe and qualified by FCM to determine the intracellular ROS level. As shown in Fig. 1A, B, the intracellular ROS level in the PA-treated group was significantly increased compared with the control group. However, DHM notably suppressed PA-induced increase of intracellular ROS. HepG2 cells were then labeled with MitoSOX™ Red to determine the mtROS levels. As shown in Fig. 1C, D, DHM treatment dominantly inhibited PA-induced mitochondria-derived ROS level in HepG2 cells. The MMP is a crucial parameter for the evaluation of mitochondrial function, which was usually detected using JC-1 fluorescent probe. As shown in Fig. 1E, F, DHM treatment significantly inhibited PA-induced decrease of MMP in HepG2 cells. Moreover, DHM treatment suppressed the increase of intracellular ROS (Additional file 1: Fig. S1A, B) and mtROS (Additional file 1: Fig. S1C, D) as well as decreased MMP (Additional file 1: Fig. S1E, F) in PA-induced HHL-5 cells.

The normal structure and function of mitochondria are closely related to redox homeostasis [30]. Filamentous and tubular mitochondria are widely agreed to be healthy, while mitochondria that are fragmented and spherical in appearance are considered to be structurally and functionally abnormal [30]. In the study, HepG2 cells were labeled with MitoTracker Red to evaluate the changes of mitochondrial structure (Fig. 1G). In PA-induced HepG2 cells, the mitochondria seemed mostly fragmented or spherical in appearance, suggesting a certain degree of dysfunction. However, when cells were pretreated with DHM, the mitochondria were considerably improved, with a notably tubular shape. The results indicated that DHM treatment resulted in an improvement in mitochondrial structure and function in PA-treated hepatocytes. To further characterize the mitochondrial ultrastructure, cells were detected by TEM. As shown in Fig. 1H, the mitochondria appeared to be swollen and round-shaped with disrupted mitochondrial cristae (red arrow) in PA-treated HepG2 cells. Whereas, DHM-treated HepG2 cells showed improved mitochondrial ultrastructure with relatively less swollen and more organized mitochondrial cristae (Fig. 1H). Interestingly, the formation of autophagosome (blue arrow) was observed in the DHM-pretreated HepG2 cells, suggesting a dominant induction of autophagy by DHM treatment. Moreover, DHM treatment resulted in an obviously increased percentage of normal mitochondria compared to PA-induced hepatocytes, implying its role in the prevention against PA-induced injury in hepatocytes (Fig. 1I). Moreover, the expression of OPA1, which is responsible for the regulation of mitochondrial fusion, was measured. It was found that PA-induced a lowered OPA1 expression in hepatocytes, which was significantly inhibited by DHM treatment (Additional file 2: Fig. S2). Overall, these results indicate that DHM could inhibit PA-induced oxidative stress and maintain normal mitochondrial structure in PA-induced hepatocytes.

### DHM suppresses PA-induced oxidative stress by induction of autophagy in hepatocytes

**Fig. 2:**
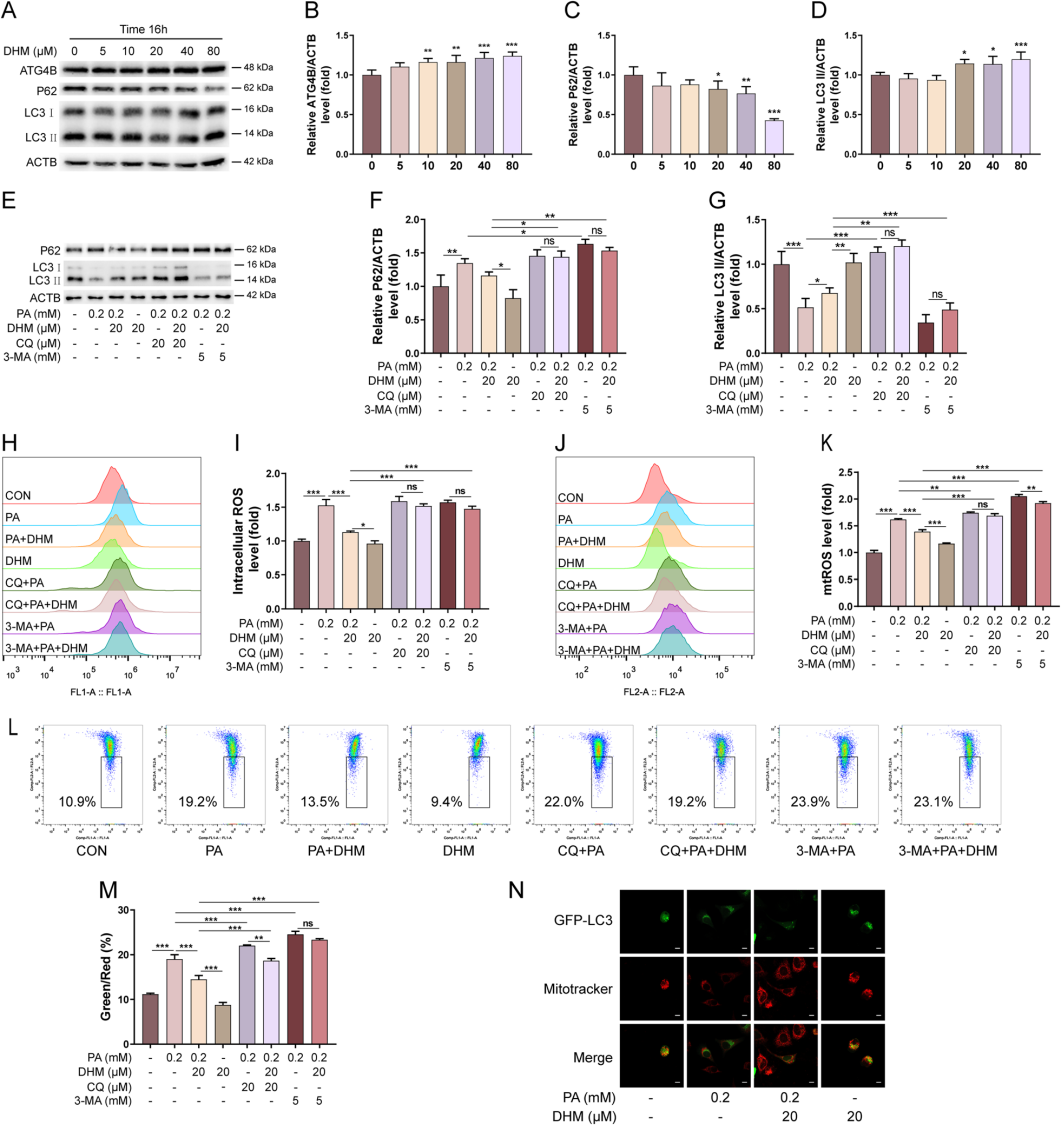
DHM suppresses PA-induced oxidative stress by induction of autophagy in hepatocytes. **A**–**D** HepG2 cells were treated with DHM of different concentrations (0, 5, 10, 20, 40 and 80 μM) for 16 h. The expressions of ATG4B, P62, and LC3 were analyzed by western blotting. Representative photographs (**A**) and densitometric quantification of ATG4B (**B**), P62 (**C**) and LC3 (**D**). **E**–**G** HepG2 cells were pretreated with CQ (20 μM) or 3-MA (5 mM) for 1 h and then treated with DHM (20 μM) for 2 h followed by exposure to PA (0.2 mM) for 16 h. The expressions of P62 and LC3 were analyzed by western blotting (**E**). The bar graphs showed the quantification of P62 (**F**) and LC3 (**G**), respectively. **H**, **I** HepG2 cells were treated as indicated. The intracellular ROS level was analyzed by FCM assay (**H**), and the bar graph showed the quantification. **J**, **K** HepG2 cells were treated as indicated. The mtROS were analyzed by FCM assay (**J**) and the bar graph showed the quantification (**K**). **L**, **M** The MMP levels were analyzed by FCM assay (**L**) and the bar graph showed the quantification (**M**). **N** HepG2 cells were transfected with the GFP-LC3 expression vector for 4 h, followed by the treatment with DHM (20 μM) for 2 h and 0.2 mM of PA for an additional 16 h. Representative images of cells co-expressing MitoTracker Red and GFP-LC3 were showed by confocal microscopy; scale bars: 10 mm. Graphs showed mean ± SEM; n = 3; data were from one experiment out of three. *p < 0.05, **p < 0.01, ***p < 0.001, compared between the marked groups; *ns* no significant difference

Previous studies reported that the occurrence of NAFLD involves with mitochondrial dysfunction of hepatocytes, which is associated with autophagy arrest [31]. To determine whether DHM has an impact on autophagy in PA-induced hepatocytes, HepG2 cells were exposed to different concentrations (5, 10, 20, 40, 80 μM) of DHM alone for 16 h. As shown in Fig. 2A, B, the protein expression of ATG4B was significantly upregulated as the increased concentrations of DHM. Moreover, treatment with DHM at a higher concentration (≥ 20 μM) resulted in a decreased expression of P62 and an increased expression of LC3 II (Fig. 2A, C, D), suggesting that DHM alone could promote autophagic flux in hepatocytes. To further investigate the influence of DHM on autophagy in PA-induced hepatocytes, autophagy inhibitors of CQ (20 µM) and 3‐MA (5 mM) were applied as indicated (Fig. 2E, G). As shown in Fig. 2E, G, PA treatment induced increased P62 and decreased LC3 II levels, and these effects were significantly suppressed by DHM treatment. However, the inhibitory effects of DHM on PA-induced autophagic flux decrease were substantially suppressed by the addition of 3-MA and CQ separately. Furthermore, DHM significantly inhibited PA-induced intracellular ROS and mtROS levels in HepG2 cells, as shown in Fig. 2H–K. However, pretreatment with 3‐MA and CQ dominantly eliminated DHM-induced amelioration on the oxidative stress in HepG2 cells (Fig. 2H–K). DHM-induced amelioration of MMP in PA-treated hepatocytes was also blocked by the addition of 3-MA and CQ separately (Fig. 2L, M). Besides, pretreatment with DHM resulted in a significant increase of GFP-LC3 puncta in HepG2 cells (Fig. 2N). Taken together, these findings demonstrate that DHM inhibits PA-induced oxidative stress by induction of autophagy in hepatocytes.

### DHM attenuates PA-induced autophagy arrest and oxidative stress in hepatocytes via SIRT3

**Fig. 3:**
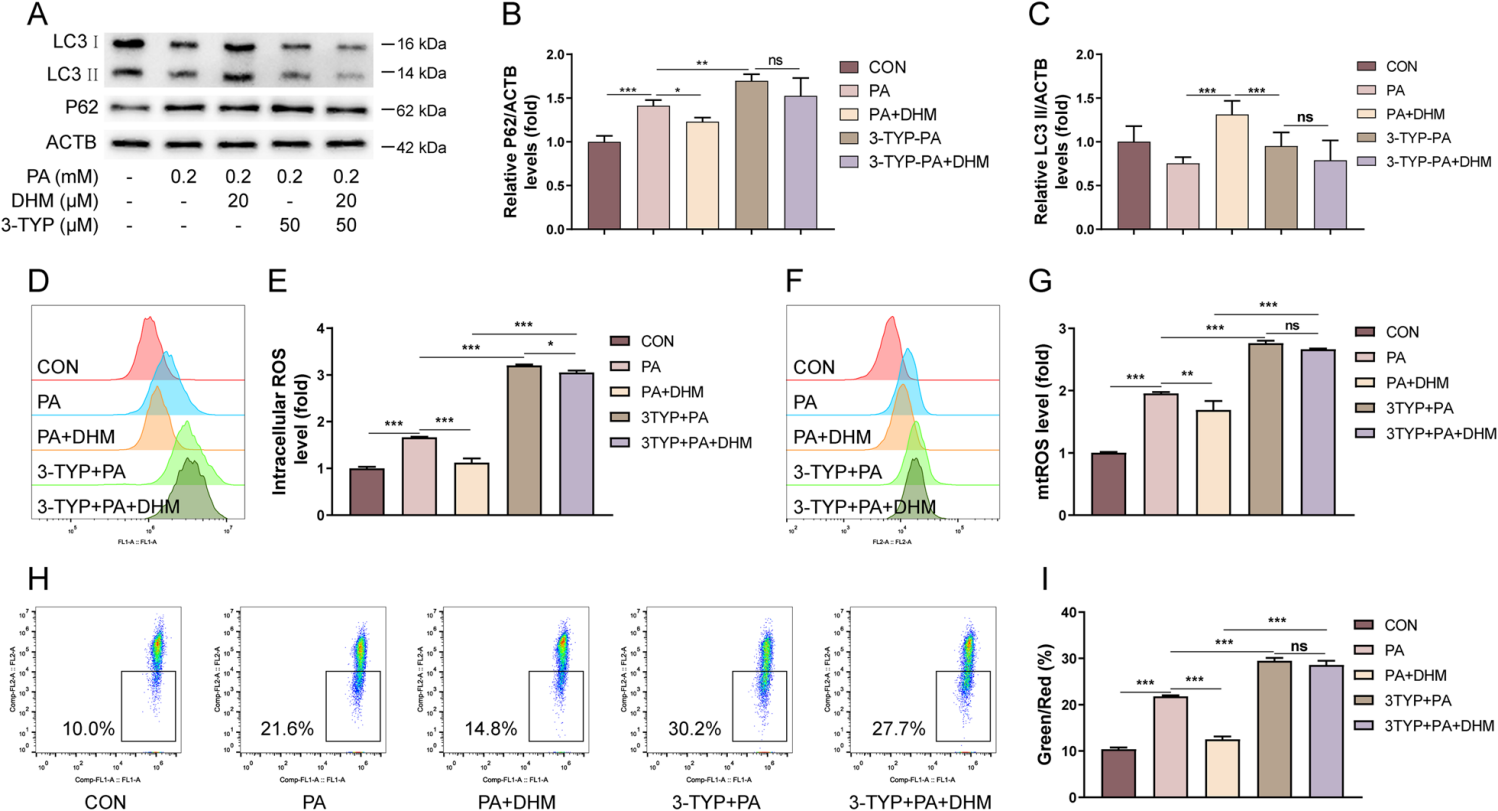
DHM attenuates PA-induced autophagy arrest and oxidative stress in hepatocytes via SIRT3. HepG2 cells were pretreated with 3-TYP (50 μM) or the vehicle for 1 h, followed by the treatment with DHM (20 μM) for 2 h and 0.2 mM of PA for 16 h. **A**–**C** Western blotting analysis of P62 and LC3 (**A**), and the densitometric quantification of P62 (**B**) and LC3 (**C**). **D**, **E** The intracellular ROS levels were measured by FCM assay (**D**), and the bar chart showed the quantification (**E**). **F**, **G** The mtROS levels were measured by FCM assay (**F**), and the bar chart showed the quantification (**G**). **H**, **I** The MMP levels were measured by FCM assay (**H**) and the bar chart showed the quantification (**I**). Graphs showed mean ± SEM; n = 3; data were from one experiment out of three. *p < 0.05, **p < 0.01, ***p < 0.001, compared between the marked groups; *ns* no significant difference

It was demonstrated that SIRT3 overexpression is involved in the activation of autophagy and mitophagy in myocytes [32, 33]. SIRT3 was also found to be a key molecule responsible for the suppression of intracellular ROS level by deacetylation of mitochondrial enzymes [34]. To determine whether SIRT3 is involved with the inhibitory effects of DHM on PA-induced autophagy arrest and oxidative stress in hepatocytes, the SIRT3 inhibitor 3-TYP was used as indicated. As shown in Fig. 3A–C, western blotting analysis indicated that the protein expression of P62 was increased and that of LC3 II was decreased in PA-treated hepatocytes, implying that PA notably suppressed autophagy in HepG2 cells. However, PA-induced autophagy inhibition was significantly attenuated by DHM treatment, and this effect was predominantly eliminated by the addition of 3-TYP (Fig. 3A–C). These results suggested that SIRT3 was involved in the regulation of autophagic flux in DHM-treated HepG2 cells. Moreover, DHM treatment suppressed the increase of intracellular ROS (Fig. 3D, E) and mtROS (Fig. 3F, G) as well as decreased MMP (Fig. 3H, I) in PA-induced hepatocytes. However, these effects were abolished by a SIRT3 inhibitor 3-TYP pretreatment. These findings indicate that DHM treatment attenuates PA-induced autophagy arrest and oxidative stress in hepatocytes, which was mediated via SIRT3.

### DHM attenuates PA-induced autophagy arrest and oxidative stress in hepatocytes partially through ATG4B

**Fig. 4:**
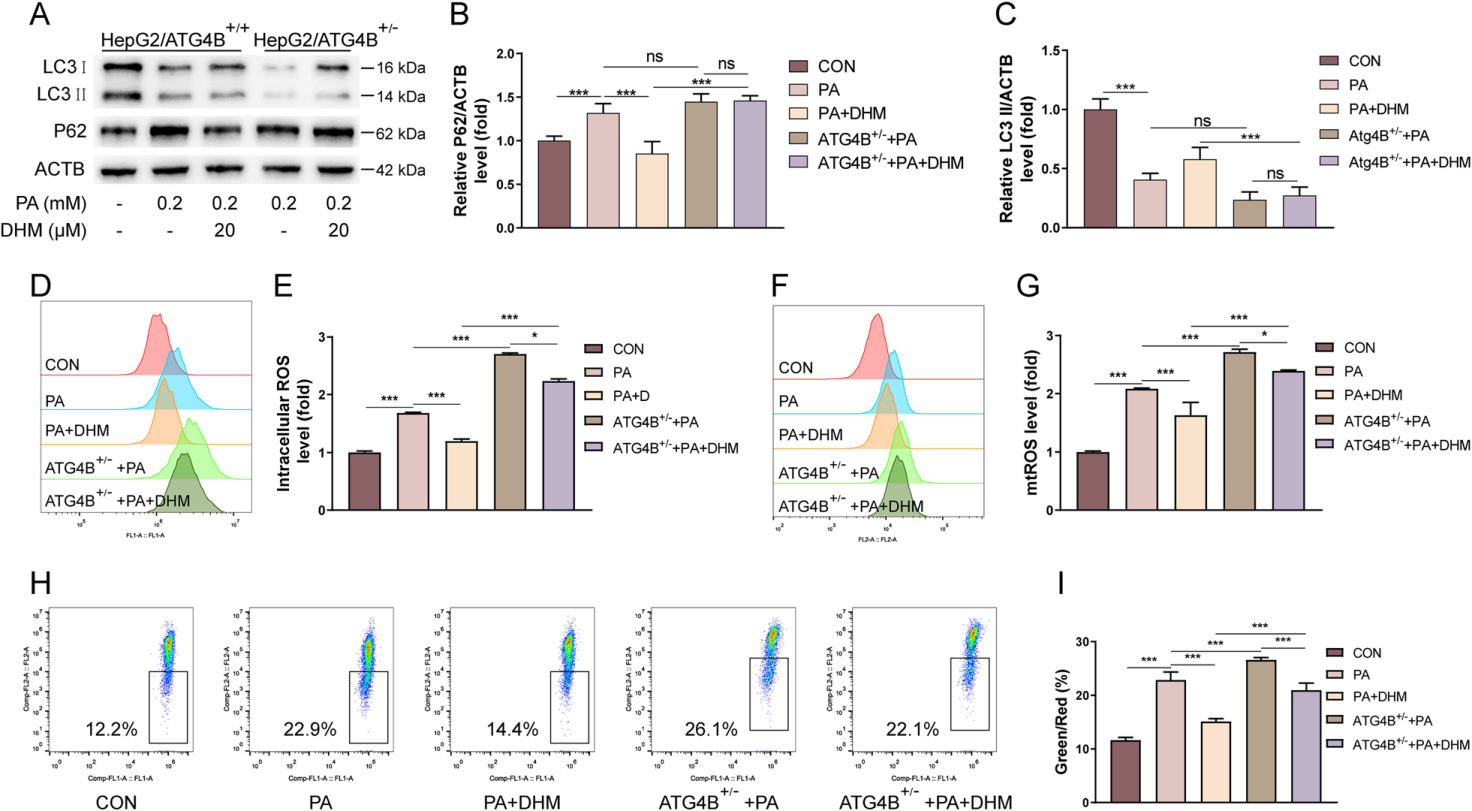
DHM attenuates PA-induced autophagy arrest and oxidative stress in hepatocytes partially through ATG4B. The HepG2 cells with ATG4B expression (HepG2/ATG4B^+/+^) and silencing ATG4B (HepG2/ATG4B^+/−^) were treated as indicated. **A**–**C** The expression of P62 and LC3 were analyzed by western blotting (**A**). And the bar graphs showed the quantification of P62 (**B**) and LC3 (**C**). **D**, **E** The intracellular ROS levels were measured by FCM assay (**D**) and quantified (**E**). **F**, **G** The mtROS levels were analyzed by FCM (**F**) and quantified (**G**). **H**, **I** The MMP levels were measured by FCM assay (**H**) and quantified (**I**). Graphs showed mean ± SEM; n = 3; data were from one experiment out of three. *p < 0.05, **p < 0.01, ***p < 0.001, compared between the marked groups; *ns* no significant difference

ATG4B is a cysteine protease that plays a significant role in the regulation of autophagy process [35]. Previous findings indicated that DHM treatment alone could upregulate the expression of ATG4B in HepG2 cells (Fig. 2A, B). Therefore, we suspected that ATG4B might also be involved in DHM-mediated modulation of autophagy and oxidative stress in PA-induced hepatocytes. We conducted experiments using HepG2/ATG4B^±^ cells with heterozygous knockout of *ATG4B* in HepG2 cell line (Additional file 3: Fig. S3). Our data indicated that DHM treatment resulted in a decreased P62 and increased LC3 II levels in HepG2 cells (Fig. 2A–C). However, these effects were blocked in HepG2/ATG4B^±^ cells, suggesting that ATG4B was essential for DHM-induced autophagy in hepatocytes (Fig. 4A–C). Similarly, DHM suppressed the increase of intracellular ROS (Fig. 4D, E) and mtROS (Fig. 4F, G) levels as well as loss of MMP (Fig. 4H, I) in HepG2 cells, which were abolished in HepG2/ATG4B^±^ cells, suggesting that ATG4B was essential for DHM-induced inhibition of oxidative stress in hepatocytes. In conclusion, these results indicate that DHM suppressed PA-induced autophagy arrest and oxidative stress in hepatocytes, which might be mediated partially through ATG4B.

### DHM facilitates SIRT3-mediated deacetylation of ATG4B in hepatocytes

**Fig. 5:**
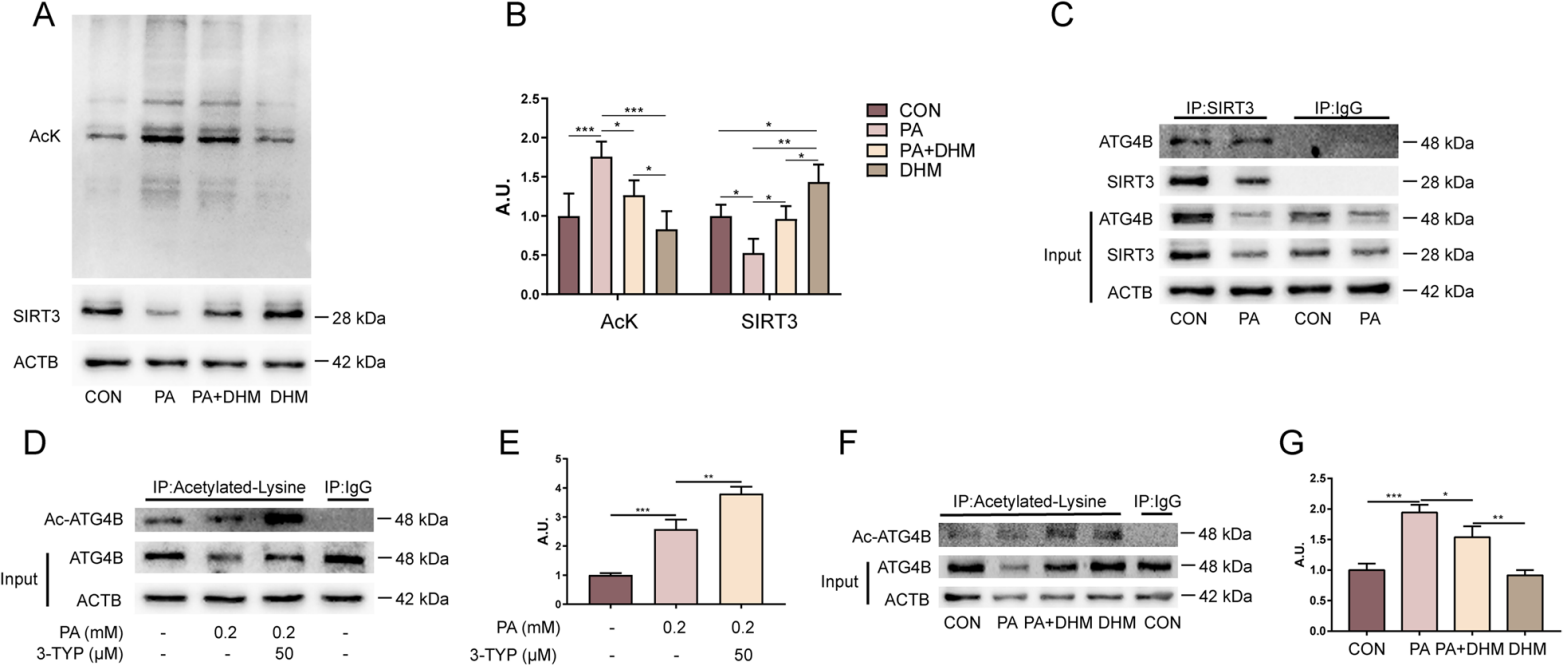
DHM facilitates SIRT3-mediated deacetylation of ATG4B in hepatocytes. **A**, **B** Western blotting analysis of total protein acetylation using an anti-acetyllysine antibody (AcK) and the protein expression of SIRT3 (**A**). The bar chart showed the quantification (**B**). **C** The expression of ATG4B followed by co-immunoprecipitated with the anti-SIRT3 antibody was analyzed by western blotting. **D**, **E** HepG2 cells were pretreated with 3-TYP (50 μM) and then treated with PA (0.2 mM). The expressions of the acetylated and total ATG4B in HepG2 cells were measured by western blotting (**D**). The densitometric quantification of the ratio of acetylated-ATG4B/ATG4B (**E**). **F**, **G** HepG2 cells were treated with DHM and followed by PA treatment. The expressions of the acetylated and total ATG4B in HepG2 cells were analyzed by western blotting (**F**). The bar graph showed the quantification of the ratio of acetylated-ATG4B/ATG4B (**G**). Graphs showed mean ± SEM; n = 3; data were from one experiment out of three. *p < 0.05, **p < 0.01, ***p < 0.001, compared between the marked groups; *A.U.* arbitrary units

Based on the findings that SITR3 and ATG4B might play crucial roles in DHM-mediated amelioration of oxidative stress in hepatocytes, the underlying mechanisms need to be further clarified. We assumed that DHM could promote autophagy through SIRT3-mediated deacetylation of ATG4B in HepG2 cells. As shown in Fig. 5A, B, DHM treatment significantly inhibited PA-induced hyperacetylation in HepG2 cells, accompanied by increased SIRT3 expression, implying a critical role of SIRT3 in DHM-induced deacetylation (Fig. 5A, B). Furthermore, it was reported that the N-terminus of ATG4B could be post-translational modified by acetylation [15]. As SIRT3 has robust deacetylase activity in mitochondria [16], we hypothesized that SIRT3 could modulate the deacetylation of ATG4B. As shown in Fig. 5C, SIRT3 could be co-immunoprecipitated with ATG4B by the Co-IP assay. Next, we sought to investigate whether ATG4B could be modulated by SIRT3-mediated deacetylation in HepG2 cells. As shown in Fig. 5D, E, the immunoprecipitated ATG4B was incubated with a specific antibody against acetyllysine residues; it was indicated that PA treatment led to increased acetylation of ATG4B in hepatocytes, which was enhanced by a SIRT3 inhibitor 3-TYP pretreatment. Moreover, DHM treatment inhibited PA-induced increase of ATG4B acetylation in HepG2 cells, as shown in Fig. 5F, G. In conclusion, DHM facilitates SIRT3-mediated deacetylation of ATG4B in hepatocytes.

## Discussion

**Fig. 6:**
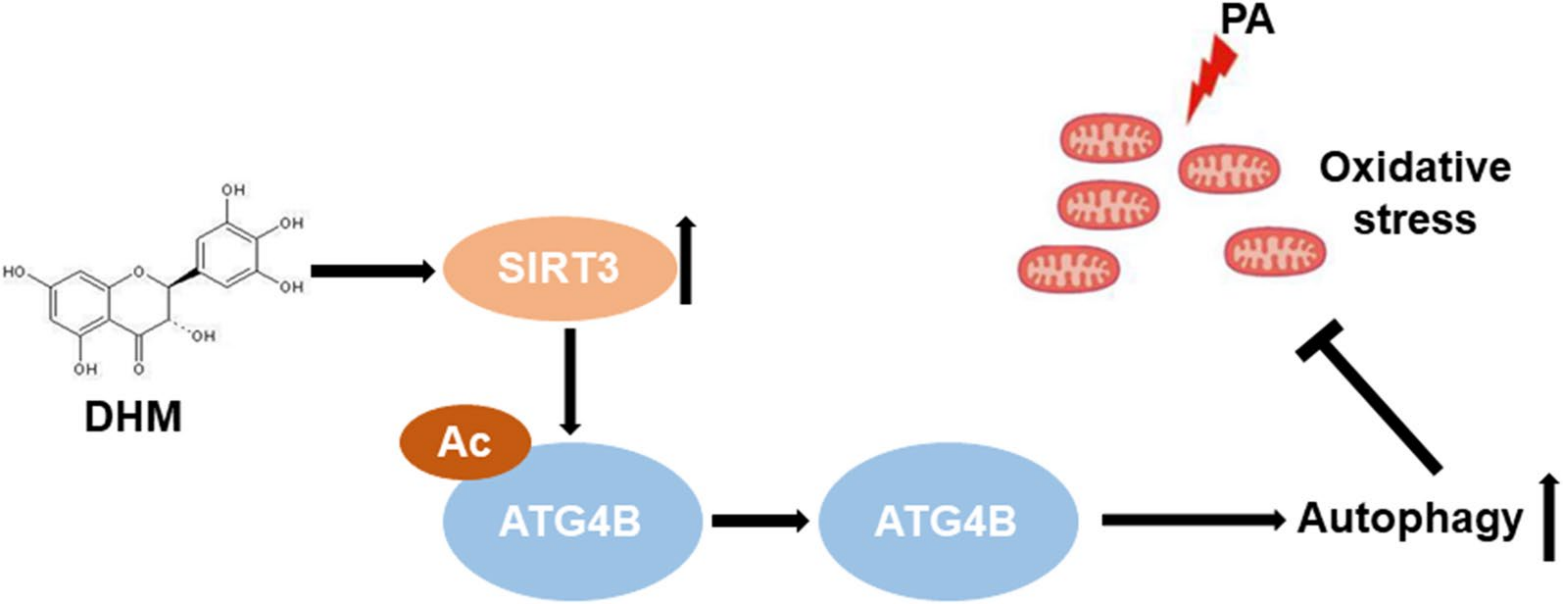
Schematic diagram of DHM-mediated amelioration of oxidative stress through induction of autophagy via a SIRT3–ATG4B signaling pathway in PA-treated hepatocytes. DHM attenuates PA-induced oxidative stress in hepatocytes through induction of autophagy, which is mediated through the increased expression of SIRT3 and SIRT3-mediated ATG4B deacetylation following DHM treatment

NAFLD is characterized by a broad spectrum of liver disorders, ranging from simple steatosis in hepatocytes to NASH, liver fibrosis and cirrhosis, and has been the most common cause of chronic liver disease in western countries [1]. According to the classical “two-hit theory”, hepatic fat accumulation (the “first hit”) followed by the elevated output of oxidative stress (the “second hit”) leads to exaggerated inflammation in the liver, eventually leading to NASH and fibrosis [3]. At present, there are no approved pharmacological treatments due to significant issues with efficacy and long-term safety [11]. Thus, dietary strategies for the prevention and treatment of NAFLD are strongly recommended. Accumulating evidence indicates that several polyphenols contribute to the enhancement of lipid accumulation and oxidative stress of the liver, and are considered to be associated with a low prevalence of metabolic diseases, including insulin resistance, hypertension, and NASH [36–38]. Thus, encouragement of consumption of polyphenol-rich diets is beneficial for the prevention and treatment of NASH. Our previous research has demonstrated that DHM, as a major flavonoid in *Ampelopsis grossedentata*, was effective at improving lipid metabolism in NAFLD patients [23]. And DHM could effectively ameliorate PA-induced steatosis in hepatocytes through improving mitochondrial respiratory chain function and antioxidant capacity via SIRT3 [24]. However, the effects of DHM on oxidative stress in hepatocytes and the underlying mechanisms have not been fully clarified. In this study, we found that DHM significantly attenuated PA-induced oxidative stress in hepatocytes through induction of autophagy, and the benefits were mediated through the deacetylation of ATG4B via increased SIRT3 expression (Fig. 6). Our results provided new insights into a SIRT3-mediated mechanism involving DHM-induced amelioration of oxidative stress in hepatocytes, indicating that SIRT3/ATG4B-mediated induction of autophagy might play an essential role in the attenuation of oxidative stress in hepatocytes.

Various studies have indicated that the mechanisms involved in the progression of NASH were related to increased ROS and oxidative stress [11, 39, 40]. Pro-oxidant systems, including cyclooxygenase, lipoxygenase, cytochrome P450, and free radical products, have been implicated, individually or cooperatively, as being involved in the production of oxidative stress in NASH. More importantly, mitochondria are critically important in ROS overproduction, which leads to mitochondrial dysfunction and abnormal respiration, culminating in NAFLD occurrence and development [11]. In the current study, we found that DHM treatment decreased the intracellular and mitochondrial ROS levels in hepatocytes, which was associated with improved mitochondrial structure and function, indicating a crucial role of DHM in the maintenance of liver redox homeostasis.

Autophagy is a conserved cellular process that affects organisms as diverse as worms, flies, yeast, and mammals [9]. The best-characterized functions of autophagy include provision of an alternative source of energy during starvation and maintenance of cellular quality control [41]. There is accumulating evidence demonstrating that autophagy may protect against steatosis and promote hepatocyte regeneration by limiting hepatocyte injury and reducing M1 polarization [42, 43]. Previous research has indicated that DHM can induce autophagy in HepG2 cells [27]. Here, we demonstrated that DHM treatment inhibited the PA-induced autophagic flux decrease in hepatocytes, thereby resulting in the removal of depolarized and damaged mitochondria, finally leading to reduced oxidative stress in hepatocytes. This finding indicates that autophagy plays a significant role in DHM-induced inhibition of oxidative stress in hepatocytes.

SIRT3, localized in the mitochondria can regulate energy metabolism and metabolic homeostasis through its deacetylase activity [16]. Some studies have reported that both increased oxidative stress and damaged mitochondria were associated with decreased SIRT3 levels, and that SIRT3 deficiency leads to increased intracellular ROS levels [44–46]. We previously found that SIRT3 could be upregulated by DHM treatment, and SIRT3 enrichment could promote mitochondrial respiratory capacity and maintenance of redox homeostasis in NAFLD [24, 47]. In this study, the protective effects of DHM in PA-induced HepG2 cells were blocked by 3-TYP treatment. Our results indicate that DHM can inhibit PA-induced oxidative stress through SIRT3-mediated autophagy induction.

It is widely accepted that ATG4 cysteine protease plays a significant role in the processing of ATG8 protein during autophagy [48]. We found that the expression of ATG4B was upregulated by DHM treatment. Additionally, the protective effects of DHM on PA-induced oxidative stress as well as autophagic flux reduction were eliminated in HepG2/ATG4B^+/−^ cells, indicating that ATG4B might play a significant role in DHM-mediated oxidative stress homeostasis. Several studies have demonstrated that low levels of ROS could regulate ATG4A and ATG4B at cysteine 81 and 78, respectively [49–51]. However, we found that increased ROS levels were accompanied with a decrease in ATG4B in PA-treated hepatocytes. Further research is needed to explore the association between ROS and ATG4B in hepatocytes. Meanwhile, it is challenging to elucidate the role of SIRT3 which localized in the mitochondria in de-acetylating cytosolic protein, ATG4B. One possible explanation is during the process of mitophagy, ATG4B bound to SIRT3 and de-acetylated after recruited close to mitochondria. Meanwhile, impaired autophagy might result in an accumulation of damaged mitochondria and exacerbating mitochondrial oxidative stress. Accordingly, the use of DHM attenuated oxidative stress and promoted autophagy.

## Conclusion

In summary, our results suggest that DHM attenuates PA-induced oxidative stress in HepG2 cells. And the beneficial effects were involved with the increased expression of SIRT3 and the interaction between SIRT3 and ATG4B, thereby leading to the deacetylation of ATG4B and the induction of autophagy. Our results provide new evidence of the links among oxidative stress, autophagy and SIRT3, which are also helpful for a better understanding of DHM-mediated preventive and therapeutic effects against oxidative stress in hepatocytes during NAFLD progression.

## Supplementary Information

**Additional file 1: Fig. S1** DHM inhibits PA-induced oxidative stress in HHL-5 cell line. HHL-5 cells were pretreated with 20 μM of DHM or the vehicle (0.5% DMSO) for 2 h, followed by the treatment of 0.2 mM of PA for an additional 16 h. (A-B) HHL-5 cells were labeled with DCFH-DA probe and the intracellular ROS levels were measured by FCM assay. (C-D) HHL-5 cells were labeled with MitoSOX™ Red probe and the mitochondria ROS (mtROS) levels were quantified by FCM assay. (E-F) HHL-5 cells were labeled with JC-1 probe and the MMP level was quantified by FCM assay. Graphs showed mean ± SEM; n = 3; data were from one experiment out of three. **p < 0.01, ***p < 0.001, compared between the marked groups.

**Additional file 2: Fig. S2** DHM increases mitochondrial fusion in PA-induced HepG2 cells. HepG2 cells were pretreated with DHM (20 μM) or the vehicle for 2 h and treated with 0.2 mM of PA for 16 h. (A) Representative images of OPA1 by immunofluorescence staining, scale bars: 10 μm. (B-C) The expression of OPA1 protein was measured by western blotting (B) and densitometric quantification (C). Graphs showed mean ± SEM; n = 3; data were from one experiment out of three. *p < 0.05, **p<0.01, ***p<0.001, compared between the marked groups.

**Additional file 3: Fig. S3** The expressions of ATG4B in HepG2/ATG4B^+/+^ and HepG2/ATG4B^+/-^ cells by western blotting. (A) The expressions of ATG4B in HepG2/ATG4B^+/+^ and HepG2/ATG4B^+/-^ cells were measured by western blotting. (B) The densitometric quantification. Graphs showed mean ± SEM; n = 3; data were from one experiment out of three. ***p<0.001, compared between the marked groups.

## Data Availability

All data generated or analyzed during this study are included in this published article or are available from the corresponding author on reasonable request.

## Abbreviations

3-MA: 3-Methyladenine
3-TYP: 3-(1H-1,2,3-triazol-4-yl) pyridine
AcK: Acetyl-lysine
ACTB: Beta-actin
ATG4B: Autophagy-related 4B cysteine peptidase
Co-IP: Co-immunoprecipitation
CQ: Chloroquine
DHM: Dihydromyricetin
HepG2: Human hepatocellular cancer cell line
LC3: Light chain 3
MMP: Mitochondrial membrane potential
mtROS: Mitochondrial reactive oxygen species
NAFLD: Nonalcoholic fatty liver disease
NASH: Nonalcoholic steatohepatitis
OPA1: Optic atrophy 1 protein
PA: Palmitic acid
P62/SQSTM1: Sequestosome 1
ROS: Reactive oxygen species
SIRT3: Silent mating type information regulation 2 homolog-3
VDAC: Voltage-dependent anion channel
