# Supplementary figures and images for: Dihydromyricetin attenuates palmitic acid-induced oxidative stress by promoting autophagy via SIRT3-ATG4B signaling in hepatocytes
Source: Nutr Metab (Lond). 2021 Sep 9;18:83. doi: 10.1186/s12986-021-00612-w (PMC8428134; doi:10.1186/s12986-021-00612-w)

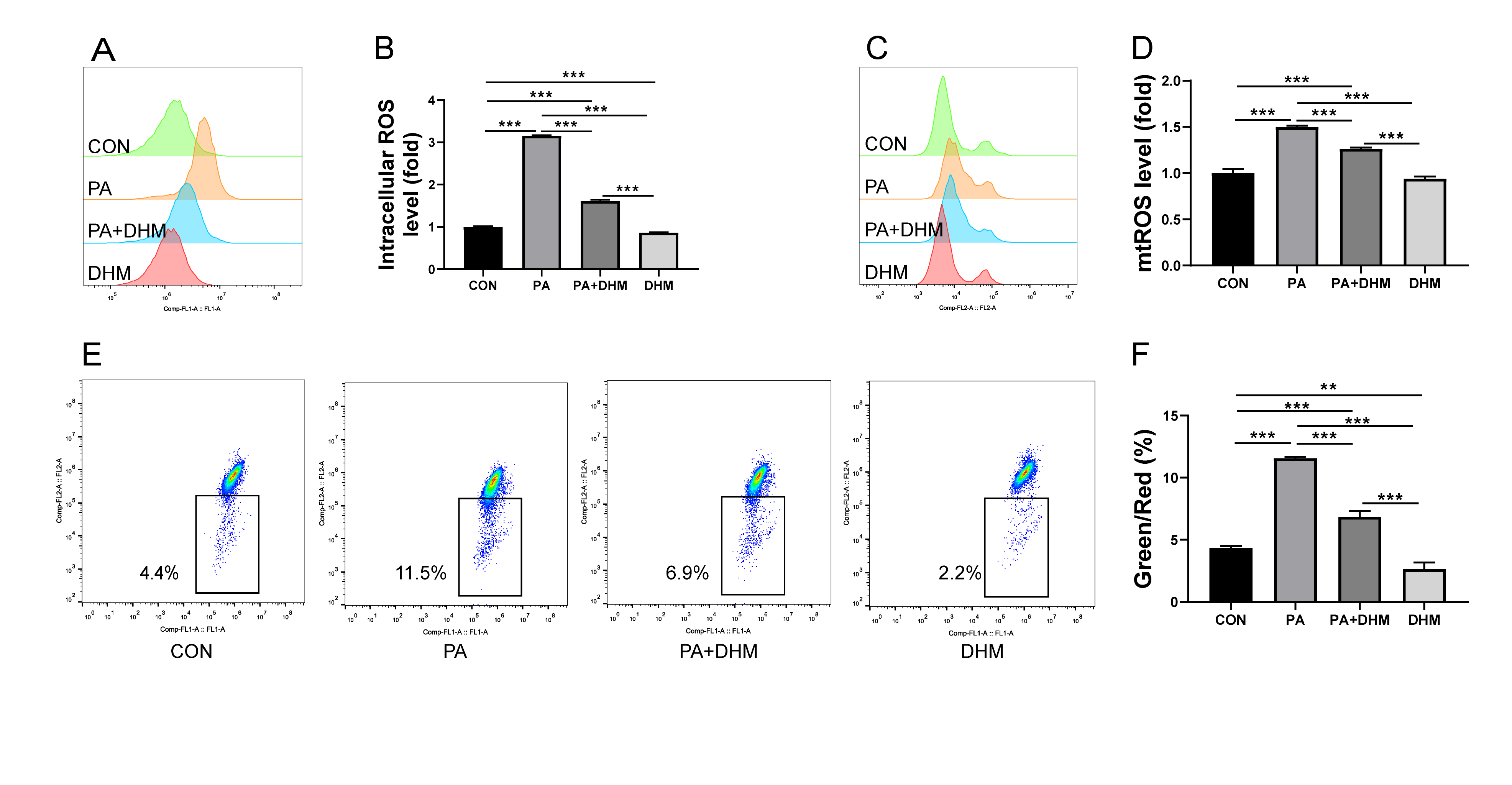

Supplement: Supplementary file 1 — Additional file 1: Fig. S1 DHM inhibits PA-induced oxidative stress in HHL-5 cell line. HHL-5 cells were pretreated with 20 μM of DHM or the vehicle (0.5% DMSO) for 2 h, followed by the treatment of 0.2 mM of PA for an additional 16 h. (A-B) HHL-5 cells were labeled with DCFH-DA probe and the intracellular ROS levels were measured by FCM assay. (C-D) HHL-5 cells were labeled with MitoSOX™ Red probe and the mitochondria ROS (mtROS) levels were quantified by FCM assay. (E-F) HHL-5 cells were labeled with JC-1 probe and the MMP level was quantified by FCM assay. Graphs showed mean ± SEM; n = 3; data were from one experiment out of three. **p < 0.01, ***p < 0.001, compared between the marked groups. [file 12986_2021_612_MOESM1_ESM.tif]

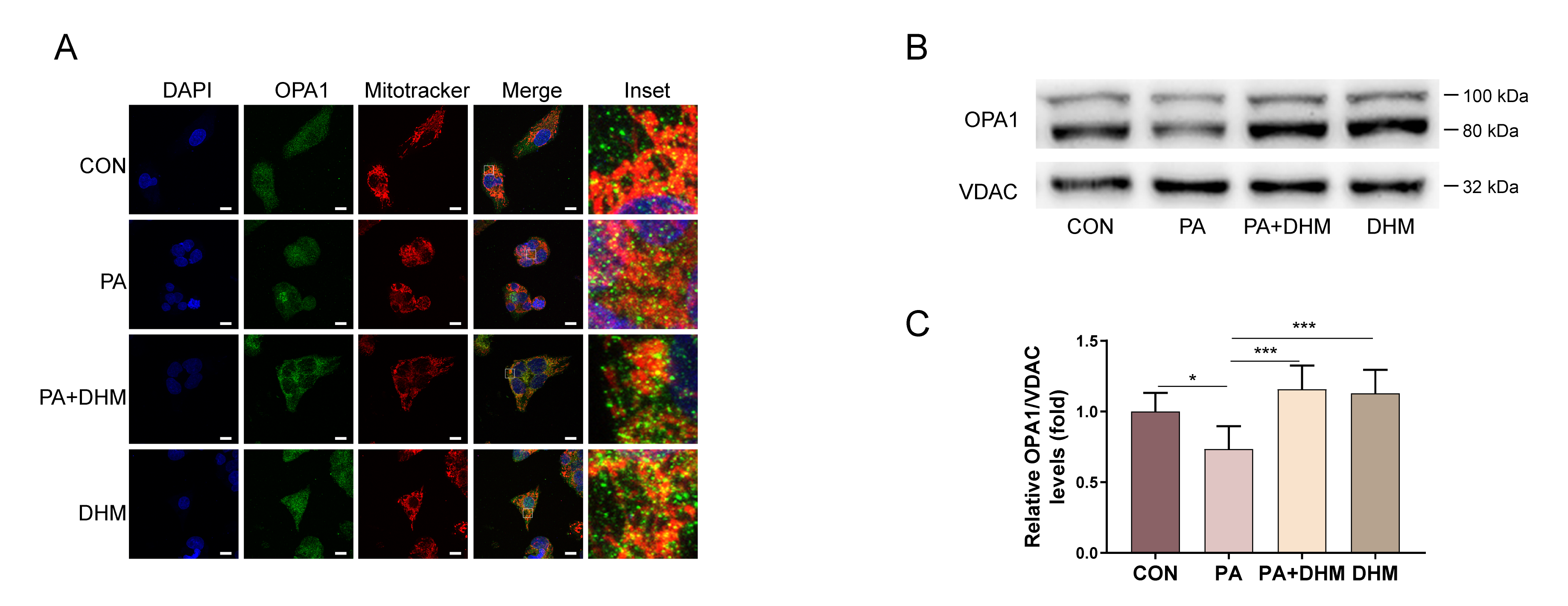

Supplement: Supplementary file 2 — Additional file 2: Fig. S2 DHM increases mitochondrial fusion in PA-induced HepG2 cells. HepG2 cells were pretreated with DHM (20 μM) or the vehicle for 2 h and treated with 0.2 mM of PA for 16 h. (A) Representative images of OPA1 by immunofluorescence staining, scale bars: 10 μm. (B-C) The expression of OPA1 protein was measured by western blotting (B) and densitometric quantification (C). Graphs showed mean ± SEM; n = 3; data were from one experiment out of three. *p < 0.05, **p<0.01, ***p<0.001, compared between the marked groups. [file 12986_2021_612_MOESM2_ESM.tif]

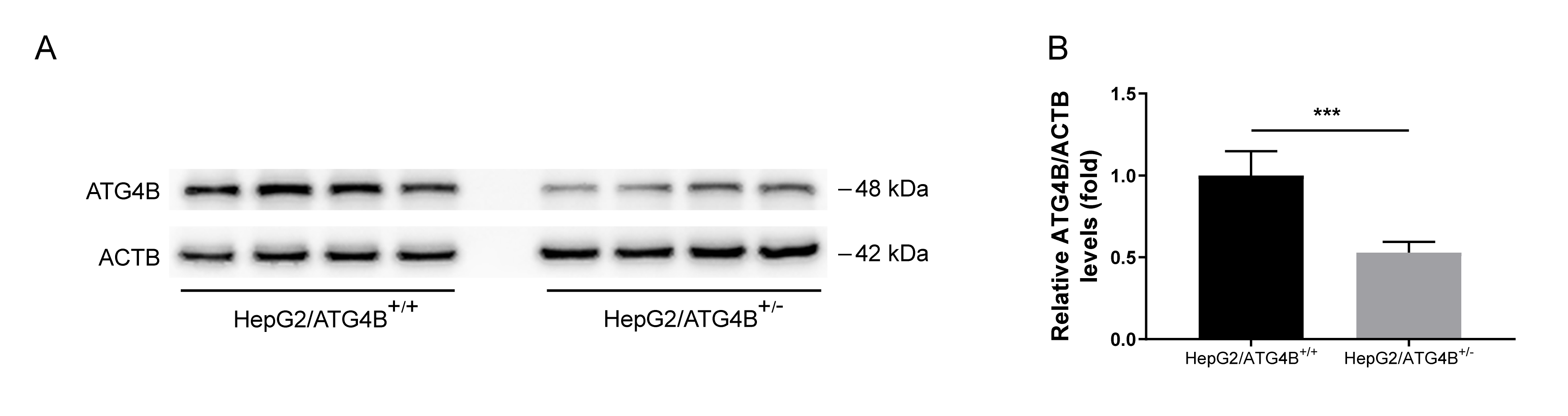

Supplement: Supplementary file 3 — Additional file 3: Fig. S3 The expressions of ATG4B in HepG2/ATG4B+/+ and HepG2/ATG4B+/- cells by western blotting. (A) The expressions of ATG4B in HepG2/ATG4B+/+ and HepG2/ATG4B+/- cells were measured by western blotting. (B) The densitometric quantification. Graphs showed mean ± SEM; n = 3; data were from one experiment out of three. ***p<0.001, compared between the marked groups. [file 12986_2021_612_MOESM3_ESM.tif]
